# The application of drones for mosquito larval habitat identification in rural environments: a practical approach for malaria control?

**DOI:** 10.1186/s12936-021-03759-2

**Published:** 2021-05-31

**Authors:** Michelle C. Stanton, Patrick Kalonde, Kennedy Zembere, Remy Hoek Spaans, Christopher M. Jones

**Affiliations:** 1 Vector Biology Department, Liverpool School of Tropical Medicine, Liverpool, UK; 2 Lancaster Medical School, Lancaster University, Lancaster, UK; 3 Malawi-Liverpool-Wellcome Trust Clinical Research Programme, Blantyre, Malawi

**Keywords:** Drones, Machine-learning, Object-based image classification, Mosquito, Anopheles, Malaria, Larval habitat, Mapping

## Abstract

**Background:**

Spatio-temporal trends in mosquito-borne diseases are driven by the locations and seasonality of larval habitat. One method of disease control is to decrease the mosquito population by modifying larval habitat, known as larval source management (LSM). In malaria control, LSM is currently considered impractical in rural areas due to perceived difficulties in identifying target areas. High resolution drone mapping is being considered as a practical solution to address this barrier. In this paper, the authors’ experiences of drone-led larval habitat identification in Malawi were used to assess the feasibility of this approach.

**Methods:**

Drone mapping and larval surveys were conducted in Kasungu district, Malawi between 2018 and 2020. Water bodies and aquatic vegetation were identified in the imagery using manual methods and geographical object-based image analysis (GeoOBIA) and the performances of the classifications were compared. Further, observations were documented on the practical aspects of capturing drone imagery for informing malaria control including cost, time, computing, and skills requirements. Larval sampling sites were characterized by biotic factors visible in drone imagery and generalized linear mixed models were used to determine their association with larval presence.

**Results:**

Imagery covering an area of 8.9 km^2^ across eight sites was captured. Larval habitat characteristics were successfully identified using GeoOBIA on images captured by a standard camera (median accuracy = 98%) with no notable improvement observed after incorporating data from a near-infrared sensor. This approach however required greater processing time and technical skills compared to manual identification. Larval samples captured from 326 sites confirmed that drone-captured characteristics, including aquatic vegetation presence and type, were significantly associated with larval presence.

**Conclusions:**

This study demonstrates the potential for drone-acquired imagery to support mosquito larval habitat identification in rural, malaria-endemic areas, although technical challenges were identified which may hinder the scale up of this approach. Potential solutions have however been identified, including strengthening linkages with the flourishing drone industry in countries such as Malawi. Further consultations are therefore needed between experts in the fields of drones, image analysis and vector control are needed to develop more detailed guidance on how this technology can be most effectively exploited in malaria control.

**Supplementary Information:**

The online version contains supplementary material available at 10.1186/s12936-021-03759-2.

## Background

Malaria cases in Africa have reduced by over half in the last two decades making transmission more heterogeneous. This has led to a growth of studies applying spatial and temporal analyses to determine where and when remaining transmission foci exist [1], and a focus on how new and existing control methods can be best utilized to reduce this residual transmission [2–4].

The geographical spread and extent of malaria transmission is limited by the seasonally-driven mosaic of water bodies available for female mosquitoes in which to lay their eggs. The ecology of preferred breeding grounds for mosquito oviposition vary both within and between species. For example, two of the main sibling species of the *Anopheles gambiae* complex, *Anopheles gambiae* and *Anopheles arabiensis*, tend to be found in transient, sunlit, small pools whereas *Anopheles funestus* is associated with more permanent, larger vegetated water [5]. At the micro-geographic scale, the presence of mosquito larvae may differ over the course of just a few metres [6–8]. Biotic and abiotic factors such as the presence of specific types of vegetation, microbiota, predators, algal density, shade, and water depth influence larval development.

Mosquito larval populations are fixed in space for the duration of their development to adulthood. Typically, eggs hatch into larvae within 2–3 days of oviposition and take 5–10 days to metamorphosize into pupae, although the speed of this process is highly dependent on temperature and the availability of nutrients [9, 10]. One method of controlling diseases transmitted by mosquitoes is to reduce the population by reducing the availability of oviposition sites and/or reduce the likelihood that resulting larvae develop into the adult stage [11]. Larval source management (LSM) involves the environmental, biological or chemical manipulation of the environment in which mosquitoes are present for the purpose of targeting the immature, aquatic stages of the mosquito and hence reducing the adult mosquito population. In the early days of mosquito control, an aggressive approach to searching and removing mosquito breeding sites was successful at reducing (and even eliminating) disease, with historical examples including its use during the construction of the Panama Canal in the early twentieth century, and its role in the elimination of *An. gambiae* in Brazil by 1940 [12]. In sub-Saharan Africa, LSM was responsible for large reductions in malaria incidence in Zambia copper mines between 1929 and 1949 [13]. LSM is, however, a labour-intensive exercise and following the introduction of IRS by DDT in the 1950s and subsequently the development of ITNs in the 1990s, it fell out of favour as a viable control option, particularly in Africa where the long rainy seasons produce countless sites for *Anopheles* development [13]. As such, LSM is currently only recommended as a complementary vector control intervention to bed nets and IRS to target residual transmission and as a method of combating insecticide resistance [14]. While its value is acknowledged by the World Health Organization (WHO) and national malaria control programmes (NMCPs) there are several barriers to its widespread implementation.

The primary barrier to implementing LSM is the issue of determining where and when the intervention should be implemented. In rural settings, the WHO recommend the application of LSM in areas where there is high coverage of long-lasting insecticidal nets (LLINs), evidence of outdoor biting and/or insecticide resistance and where larval sources are ‘*few, fixed and findable*’. Despite the lack of a clear definition of what can be considered ‘few’, ‘fixed’ or ‘findable’, this has led to many considering LSM to be impractical in rural areas with diffuse seasonal larval habitats. The perception of these terms may evolve as technology and processes for implementing LSM advance. This paper focuses on challenging the ‘*findable*’ component of this trio of conditions.

Geospatial technology is rapidly evolving and what now constitutes as ‘*findable’* may switch from less reliance on exhaustive ground-based searches to remotely sensed data. Drone mapping is being touted as at least equivalent (if not superior) to and more cost-effective than mapping larval habitat manually [15, 16] or using remotely sensed satellite imagery [17, 18]. While the latter can cover vast areas in a single day, images are often obscured by clouds and although very high-resolution commercial satellite imagery exists, the resolution (at best 30 cm) is still inferior to that obtained by drones (2–10 cm) with the time of image captured out of the data user’s control.

This paper explores the use of drones as a method for collecting very high resolution (< 10 cm), contemporary imagery of an area for the purposes of identifying larval habitat. The issues addressed include the process of capturing drone imagery (by who, how much, how often), processing the images to extract the required information (what software, image classification methods, computer processing requirements), collecting ‘ground-truth’ data (entomological sampling), and subsequently summarising this information into recommendations that can be used by the control program implementers to guide their LSM activities.

## Methods

### Drone image capturing

**Fig. 1:**
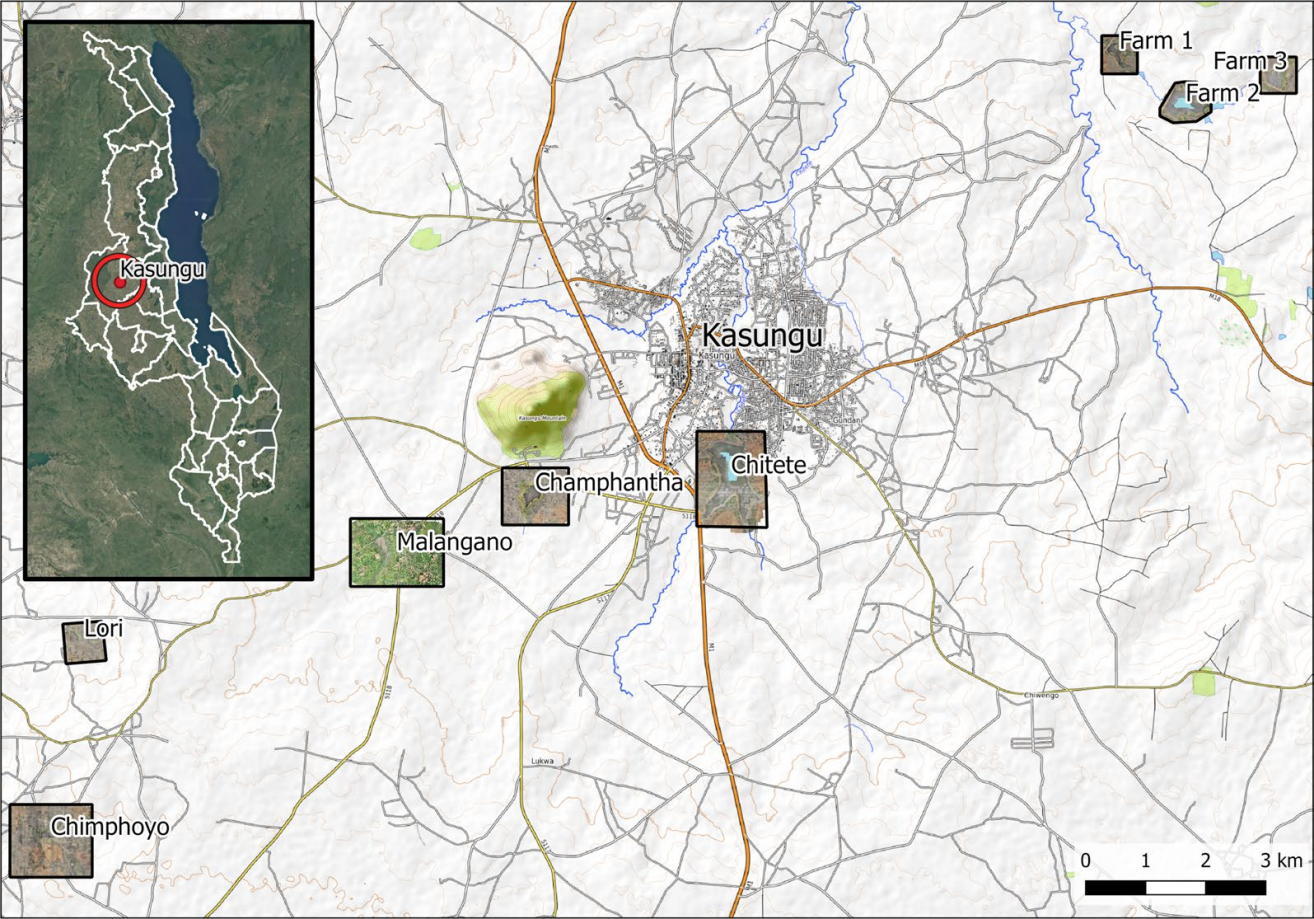
Locations of sites surveyed within the ‘humanitarian drone testing corridor’, centred on Kasungu town, Central Malawi (inset). Coordinates can be found in Additional file 1

A series of image data capture exercises were conducted within Kasungu district, central Malawi in an area that has been designated by the Government of Malawi, in collaboration with UNICEF, as a ‘humanitarian drone testing corridor’ (Fig. 1). Authorization to conduct these flights was obtained from the Malawi Department of Civil Aviation. Malaria transmission occurs all year round in this area, with parasite prevalence in children between 2 and 10 years old estimated at 19% in 2017 [19]. This transmission is potentially driven by a number of reservoirs (artificial lakes) which provide permanent sources of water within which female *Anopheles* can lay their eggs [20]. Images were captured over three visits in June 2018 (early dry season), October 2019 (late dry season) and February 2020 (wet season) using two drones, both of which were able to capture images using a standard RGB camera, plus a near-infrared (NIR) camera. Both drones were purchased off-the-shelf from commercial vendors (Table 1). The first was a multirotor (quadcopter) type aimed towards the ‘hobbyist’ market (the DJI Phantom 4 Pro), supplemented by an additional NIR sensor by Sentera [21]. The second was a fixed-wing drone marketed towards the agriculture industry (eBee SQ) which incorporated a Parrot Sequoia multispectral camera.

**Table 1:** Comparison of approximate costs required to capture and process imagery

			Phantom 4 Pro	GBP (£)	eBeeSQ	GBP (£)
Costs	Initial costs	Standard drone	RGB only	£1500	RGB only	N/A
		Supplementary sensor	Sentera NDVI	£1800	Parrot Sequoia	£7000+
	Supplementary hardware		Tablet	£150	High spec laptop	£1000+
		Spare batteries	≤ 30 min flight time (per battery)	£150	≤ 1 h flight time (per battery)	£90
	Supplementary software	Mission planning	Pix4D capture	£0	eMotion Ag	£0
		Image processing	Agisoft MetaShape Professional Edition (Educational Licence)	£425	Agisoft MetaShape Professional Edition (Educational Licence)	£425
		Image classification	Orfeo Toolbox	£0	Orfeo Toolbox	£0

### Drone image processing

Individual images captured by both drones during each mapping mission were stitched together into orthomosaics using a structure-from-motion (SfM) workflow within the commercial image processing software Agisoft Metashape Professional (version 1.4.2). SfM is a photogrammetry approach which uses multiple 2D images overlapping images to construct a 3D landscape in the form of an elevation point cloud, and subsequently geometrically correct and combine the aerial images [22]. A subset of images captured during the wet season were then classified using a geographical object-based image analysis (GeoOBIA), using the LargeScaleMeanShift algorithm within the open source software Orfeo Toolbox (OTB, version 7.1.0), applied within the QGIS environment (version 3.8.1). GeoOBIA involves grouping contiguous pixels into ‘objects’ or ‘segments’ such that each segment is relatively homogenous (within a prespecified threshold) with respect to pixel characteristics. In this instance, pixels were grouped into segments according the values of red, green, blue and elevation, with the latter being estimated using photogrammetric methods within Agisoft Metashape and then rescaled to lie between 0 and 255 to match the scale of the RGB values. Trial and error was used to select the optimal segmentation parameters i.e. the spatial radius, range radius and minimum segment size. The smoothing radius determines how the amount by which the image is smoothed or filtered prior to the segmentation algorithm being implemented, whereas the range radius determines the similarity between pixels for grouping within the same segment. Similarity in this context refers to the Euclidean distance between two pixels.

Supervised classification was then undertaken to assign each segment to one of 12 land cover classes identified in the image: open water, floating aquatic vegetation, emergent aquatic vegetation, submerged aquatic vegetation, trees/bushes, grass, bare soil, iron roof, rusted iron roof, thatched roof, tarmac road, untarmacked roads/paths (Additional file 2). Additional file 3 displays the segment-level characteristics used to train the classification algorithm, which incorporated characteristics related to segment texture (Haralick textural features [23]) in addition to a range of water and vegetation indices. The mean and variance of each of these were used in the classification.

Classification was undertaken using a set of 1800 segments all of which were firstly manually classified by the research team. One-third of the segments (n = 600) were within a 400 m by 400 m area and were used to train the classification algorithm. An additional one-third were in the same 400 m by 400 m area and were used for evaluating the accuracy of the classification within the same geographical area used for training (spatial interpolation), whereas the remaining 600 segments were distributed outside of the area used for training (spatial extrapolation). Classification was undertaken in R (version 3.6.1) using the caret package and the Random Forests (RF) classification algorithm [24]. OTB can be used to undertake the classification process, however R was used to more efficiently interrogate the data to determine the influence of training and testing set selection on classification accuracy. Therefore, whilst a wide range of supervised classification algorithms are available in caret, RF was chosen as this method is available in OTB and other commonly used commercial GeoOBIA software such as eCognition [25, 26]. Manually classified segments within the 400 m by 400 m area were randomly split into training and testing segments. A ten-fold cross validation approach was used to determine the optimal tuning parameters for the RF algorithm, and the resulting model was then applied to the testing segments. Scores were then produced to determine the accuracy of the classification for the interpolated and extrapolated testing segments considering all 12 land cover classes, followed by a reduced classification that only differentiated between surface water (open water and aquatic vegetation) and any other class. These scores included the percentage of classifications that were correct (accuracy), Cohen’s kappa agreement statistic, quantity disagreement, i.e. the difference in the number of segments classified as each category between the manual and automated classification, and allocation disagreement, i.e. difference in locations of segments classified in each category, as overall measures. Quantity and allocation disagreement were calculated using the diffeR package in R [27]. Further, the producer and user accuracy, also known as recall and precision respectively, were calculated for each of the 12 individual classes, and for the reduced classification categories. The classification was then applied to the entire 600 m by 800 m area, and the percentage of the area classed as being covered in surface water was calculated. This process of randomly splitting the segments into training and testing groups was repeated 100 times, and the median and inter-quartile range of the resulting accuracy, kappa agreement and percentage surface water cover were reported.

**Fig. 2:**
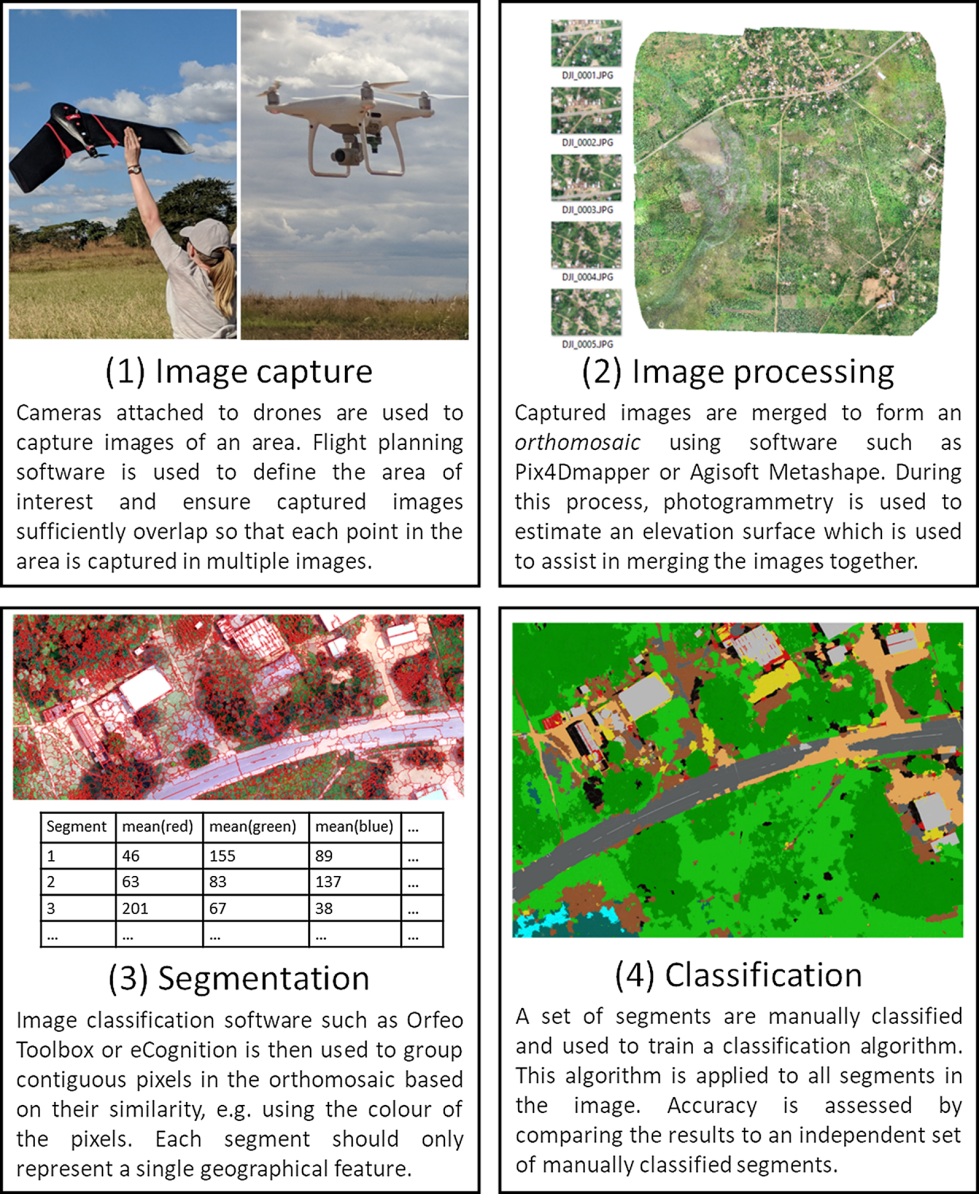
Processes undertaken to identify larval habitat from drone imagery

A manual classification of surface water was also undertaken which involved systematically scanning through the image from left to right and drawing a polygon around each area determined by the assessor to be surface water, regardless of whether it was open water or aquatic vegetation. This process was undertaken independently of the manual generation of training and testing segments, hence polygons were not constrained by the boundaries of the segments. This manual classification was undertaken by two researchers independently to evaluate the consistency of manual classifications. The union of these two areas was then considered as the manually classified surface water layer. As both the derivation of the training and testing segments plus the fully manual classification were undertaken by the same researchers and involve subjectively determining whether a segment (GeoOBIA approach) or area of pixels (manual approach) was classed as surface water, the accuracy of the two approaches cannot be objectively compared against one another. Instead, the level of agreement between the two resulting surface water polygons was evaluated by calculating the area covered by the union of the outputs obtained using the two approaches and calculating the percentage of the union covered by the intersect of the two outputs. A percentage of 100 implies the two outputs are identical whereas a value of zero implies there is complete disagreement between the two. The time taken and the computer resources required for each of these tasks were also recorded. An overview of the image capture, processing and classification procedures is presented in Fig. 2.

### Entomological sampling

Larval surveys were conducted concurrently to the drone image capture during each field visit (Fig. 1). Permission to collect these data were obtained from the Kasungu District Council, Kasungu Water Board and private landowners. Sites were selected as part of a larger study exploring the role of permanent water bodies such as local reservoirs on dry season transmission, with drone imagery being captured to monitor the changes in the landscape as the season transitioned from dry to wet. Due to delays in fieldwork as a result of Covid-19, this full study is still ongoing, and results will be published elsewhere. Preliminary work to determine the presence of mosquito larvae in the area in both wet and dry season involved three field visits. The first two field visits were undertaken during the dry season, with larval sampling being undertaken at regular intervals around the periphery of reservoirs only, with surface water being scarce elsewhere. Reservoirs were selected purposively based on their proximity to Kasungu town (for accessibility) and their proximity to human settlements. The third visit was conducted during the wet season, and sampling was focused around one of the reservoirs sampled in the previous dry season. During this period both temporary and permanent water bodies were present and larval surveys were undertaken in a selection of these sites that had been identified using drone imagery captured the previous day. A subset of sites was sampled on four consecutive days to determine their consistency with respect to larval presence.

At each site, the presence and number of larvae were recorded using 10 repeated dips of the surface water, categorised by stage (L1/L2 or L3/L4) and either anopheline or culicine. To characterise malaria vectors in Kasungu as part of the study’s broader efforts to understand transmission in the area, all anopheline larvae were raised to adult stage and identified morphologically to species [28]. The location, description and photographs of each site were recorded using an Android Smartphone and Open Data Kit (ODK). These photographs were later used by the research team to manually evaluate each site according to the amount (none, 0–1/3, 1/3−2/3, > 2/3 of the area) and type of vegetation present plus turbidity (turbid or clear). A generalized linear mixed model was then fitted to the resulting presence/absence data using R (v 3.6.3) to predict the likelihood that larvae were present from biotic site information obtained via drone imagery (vegetation type, coverage, turbidity) which were included as fixed effects, with sample area being included as a random effect. Season (wet/dry) was included as an additional fixed effect in the model. Models were fitted to presence/absence data for any larvae irrespective of stage or genus, and for late stage larvae only as characteristics of habitat containing late stage larvae are considered by WHO to be of greater importance than early stage [11]. The productivity of the sampled sites with respect to the number of early or late stage larvae collected was also considered.

## Results

### Image capturing

During the three sampling periods, images of 10 distinct areas in Kasungu were captured, covering an area of 8.9 km^2^. The two drones significantly differ in relation to operational costs, equipment and software requirements and usage. Tables 1 and 2 describe the primary differences in relation to initial costs and operational usage respectively. The fixed wing drone (eBeeSQ) had a greater initial cost than the multirotor Phantom 4 Pro due to it being inclusive of a NIR sensor, costing approximately £7000 (inclusive of an educational discount), compared to £3300 for a standard (RGB sensor) Phantom 4 Pro drone on which a NIR sensor was retrofitted. The eBeeSQ also required a high-spec laptop (£1000+) on which to run the software required to plan and conduct missions, whereas the Phantom 4 Pro was operated using free apps installed on GPS-enabled Android or iOS smartphone or tablet devices. On an operational level, the primary differences are between the flight times per battery, and the ease of use (Table 2). While, overall the Phantom 4 Pro is easier to use due to the small amount of open space required for take-off and landing, its limited battery life means that to cover a relatively modest area of 1 km^2^, 2–3 individual flights are needed depending on whether a fixed launch site is used or whether this is adapted to minimize flight time. This comes at a cost of both time and money, particularly given each battery comes at a price of £150. The eBeeSQ fixed wing drone requires less energy to fly and therefore batteries last approximately twice as long (up to 1 h in comparison to 30 min theoretical flight time, 45 min in comparison to 22 min practical flight time) than the Phantom 4 Pro. Therefore, while the time required to cover the same area is longer, this area can be comfortably covered using fewer batteries and a single launch site, meaning that in practice the process is more efficient. For example, the eBeeSQ takes 66 min (two batteries) to cover a square with an area of 1 km^2^ compared to 38 min (three batteries) with the Phantom 4 Pro when flying at 120 m above sea level (asl) with an 80% overlap in captured images. The fixed-wing drone is however more difficult to operate than the Phantom 4 Pro, requires a larger space for take-off and landing, and therefore cannot be used in more densely vegetated areas.

**Table 2:** Practical and operational differences between the drones used in this study

		Phantom 4 Pro	eBee SQ
Type of drone		Multirotor/multicopter	Fixed wing
Battery life (mins)		30	60
Practical flight time		22	45
Area (km^2^) covered per battery			
120 m asl and 80% overlap		0.49	0.64
Time (mins) required to cover 1 km^2^			
120 m asl and 80% overlap		38	66
Image resolution at 120 m asl (cm/pixel)			
RGB camera		3.3	3.7
NIR sensor		11	11
Ease of use			
Mission planning		Via app on tablet/smartphone	Via software installed on laptop computer
Take-off and landing		Vertical take-off and landing	Manual launch, and gradual descent in clear area

### Image processing and classification

**Fig. 3:**
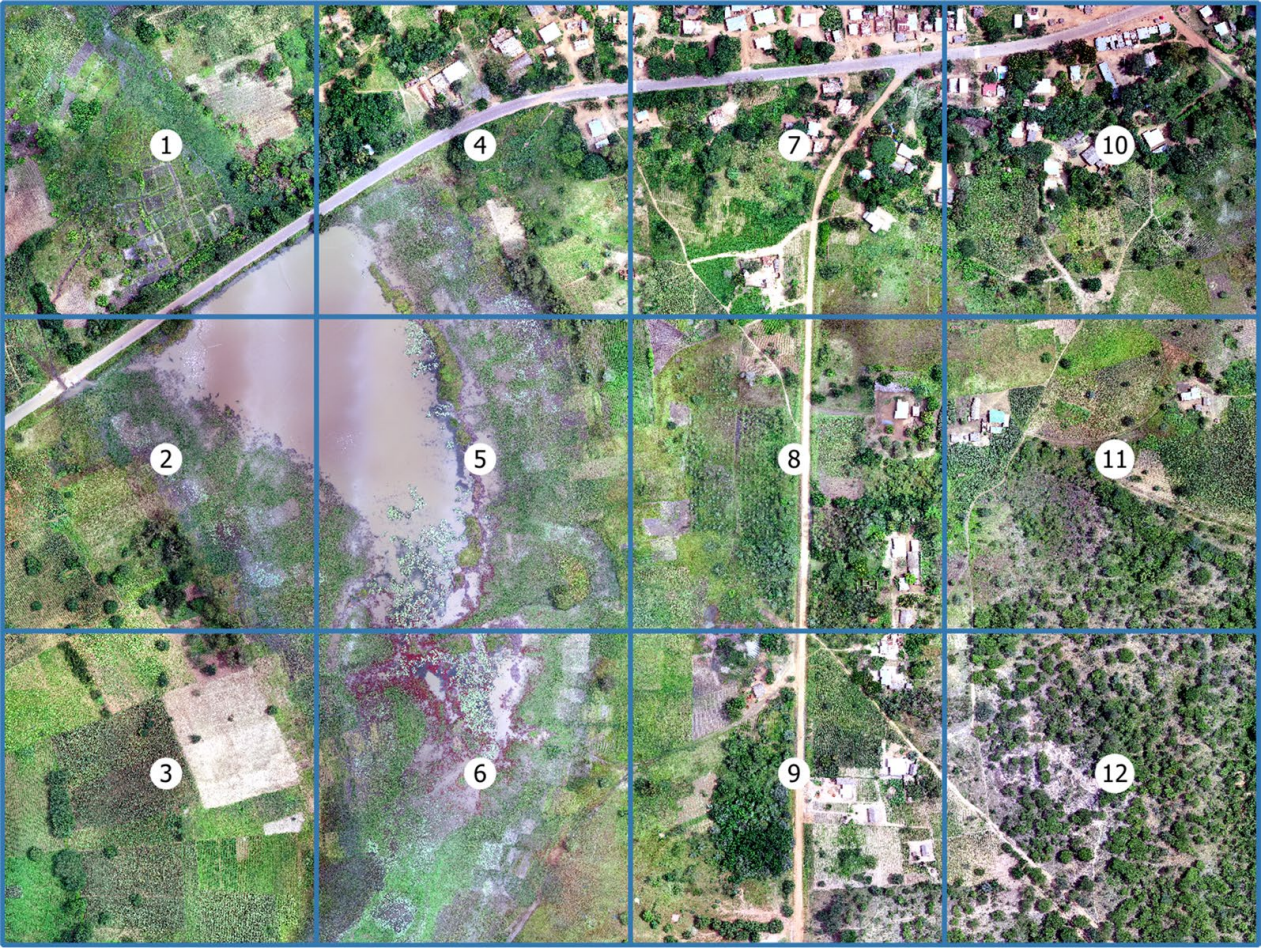
Image captured by the Phantom 4 Pro in Kasungu in February 2020 covering 800 m by 600 m, with each grid representing 200 m by 200 m. Grids 4, 5, 7 and 8 were used for training the classification algorithm, and an assessment of its accuracy was made using features both within this area (interpolation) and in the surrounding grids (extrapolation)

Agisoft Metashape was used to process all images captured. Images captured by the Phantom 4 Pro over Malangano in the wet season were used to demonstrate the processing and classification process (Fig. 1). Flying at 120 m above surface level, with an 70% overlap in images, a total of 782 individual images (6.2 GB) covered an area of 1.77 km^2^. Processing these images in Agisoft Metashape in order to produce an orthomosaic of the area and an accompanying digital surface model took a total of 250 min using a computer with an Intel Core i7-6700 processor, 32 GB RAM, resulting in an orthomosaic with a spatial resolution of 3 cm (file size = 4.2 GB). A subset of the image covering an area of 0.48 km^2^ (800 m by 600 m, file size = 1.9 GB) was then selected for classification (Fig. 3).

**Fig. 4:**
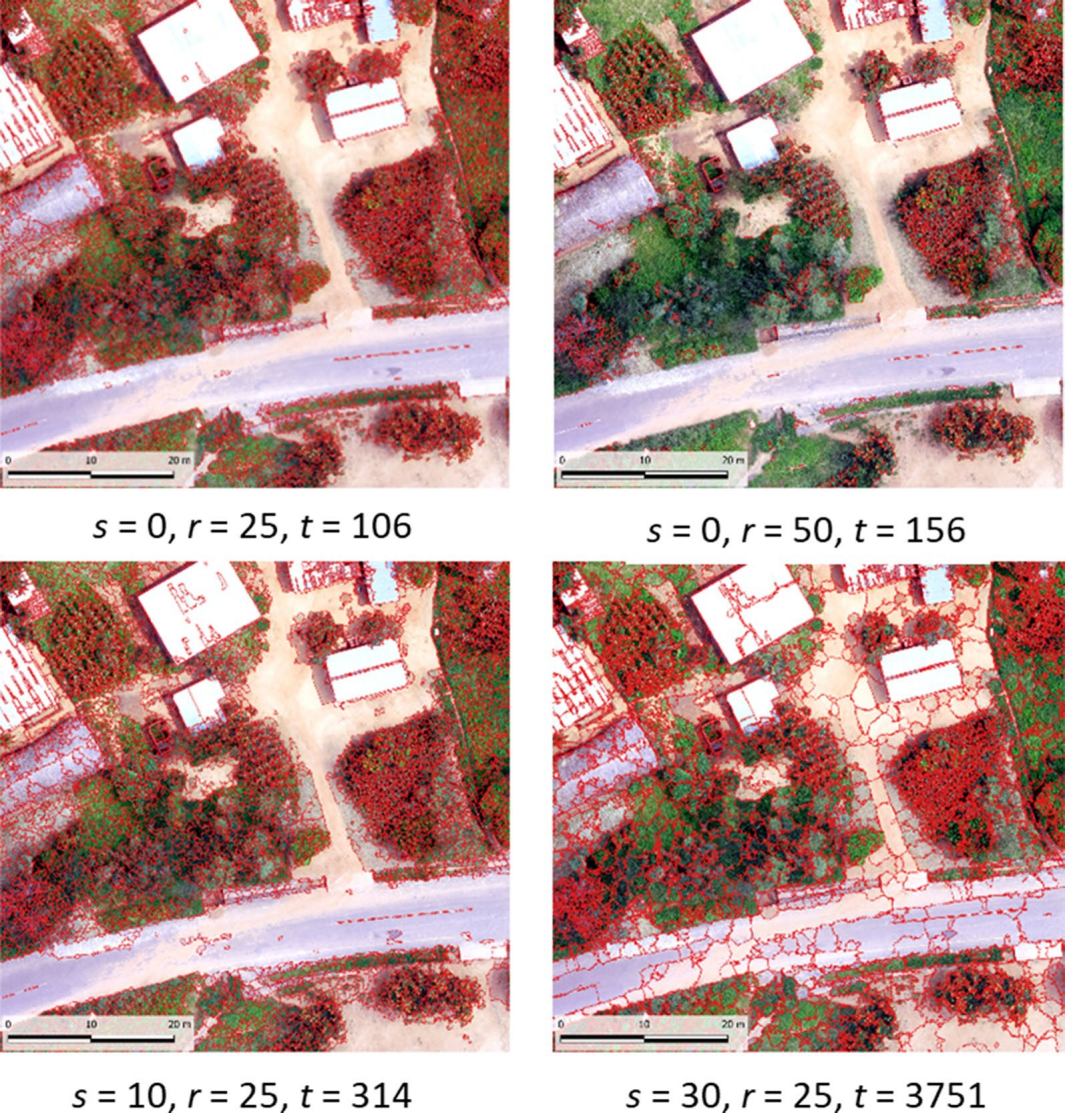
Examples of the segmentation process under different values for spatial radius *s* (0,10, 30), range radius *r* (25, 50) with a minimum segment size of 100. Time *t* corresponds to the time taken in seconds to segment a 100 m by 100 m image with a spatial resolution of 3 cm using the LargeScaleMeanShift algorithm in Orfeo Toolbox. This process includes calculating the mean and variance of the RGB and elevation values for each segment

A set of 1800 training and testing segments were then generated for the 12 identified land classes (Additional file 2), plus an additional category representing areas that were in shadow. This process took approximately six hours. The study area was partitioned into cells of 200 m by 200 m, labelled as cells 1–8 (Fig. 3), and the training and testing segments were proportionally distributed throughout the cells as follows: two thirds (1200) of segments were within cells 4, 5, 7 and 8 covering an area of 400 m by 400 m, referred to in this paper as the *internal* segments. One third of segments (600) were within the remaining cells (1–3, 6, 9, 10–12), referred to in this paper as *external* segments. Segmentation was performed using Orfeo Toolbox (OTB) functions within the QGIS environment. Figure 4 demonstrates the impact of varying values of the spatial and range radius on the resulting segmentation and the time taken to perform this segmentation over a 100 m by 100 m area using the computer specifications previously specified (see "Methods"). While increasing the spatial radius provided a more adequate balance between over-segmentation (single discrete features of interest being split into many segments) and under-segmentation (multiple discrete features of interest being grouped into a single segment), this came at the price of substantially increasing the processing time. Additional processing time is required to calculate the segment-level summaries (mean, variance) of each of the variables being used to classify the imagery, with processing time increasing as the number of segments increases.

The segmentation process was then applied to the entire 800 m by 600 m area using the parameters 10 (spatial radius), 25 (range radius) and 200 (minimum segment size), creating close to 800,000 segments. The total processing time, which includes calculating the segment-level mean and variance of the RGB and elevation values, was 24.5 h with an additional 15 h taken to calculate the mean and variance of each of the additional variables under consideration (Additional file 2). Two classifications were then undertaken, one of which included the NIR-derived variables and one of which did not.

**Fig. 5:**
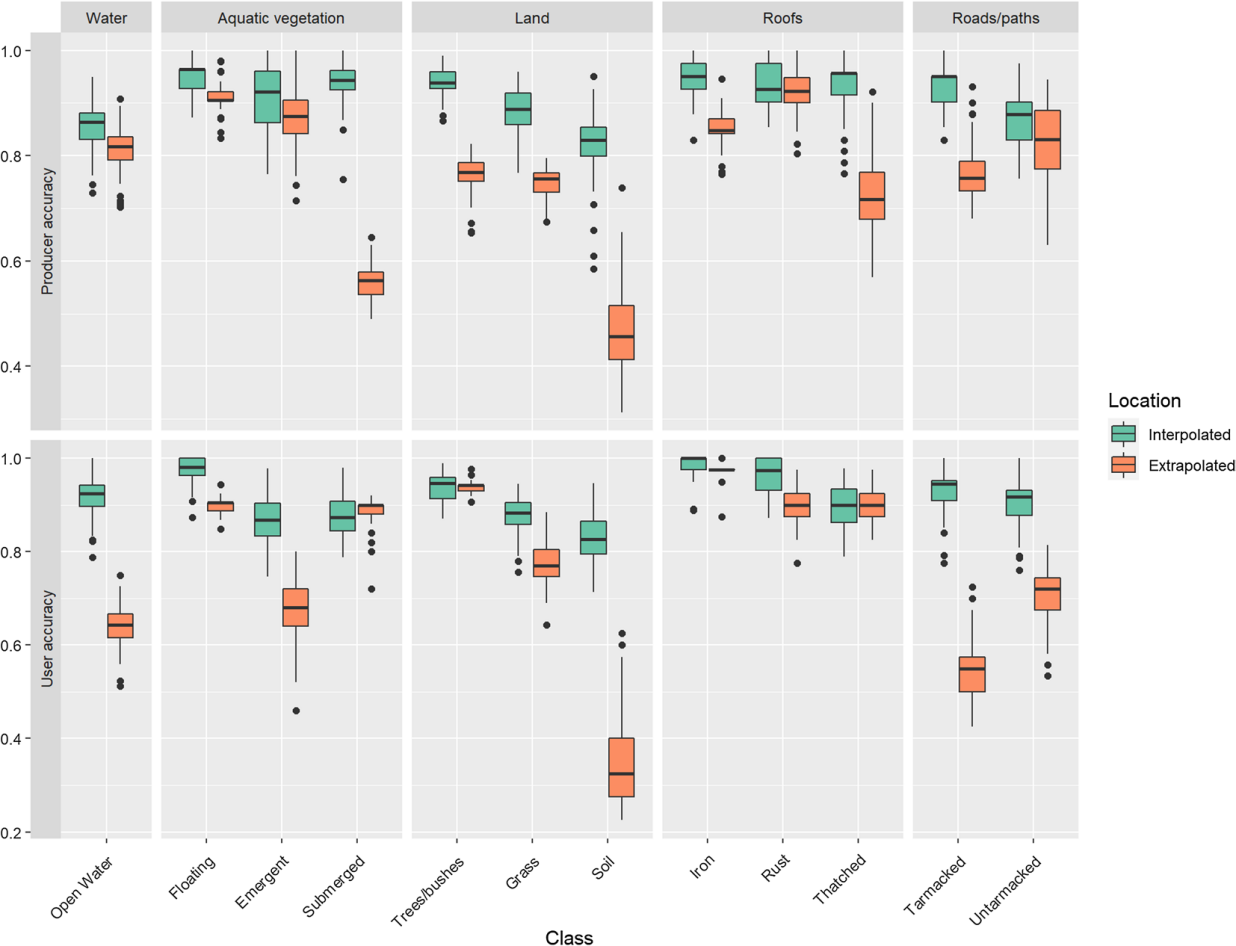
Boxplots of class-level user and producer accuracy for the classifications undertaken without using NIR-derived variables

**Fig. 6:**
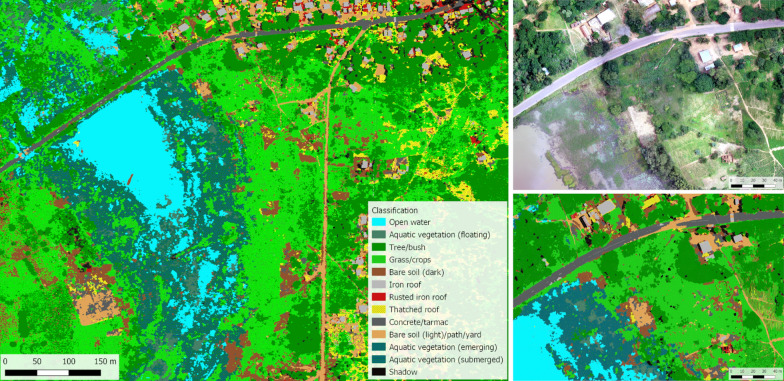
Example of a classification obtained for the entire study area using the random forests algorithm without including NIR-derived variables (left), with a more detailed view of a smaller area comparing the original image (top right) with the classified image (bottom right)

The resulting accuracy assessments of these classifications obtained without using NIR-derived variables are presented in Table 3, with class-level producer and user accuracy across all 100 samples presented in Fig. 5. For interpolated areas, user and producer accuracy was generally high (> 80%) for all classes. In extrapolated areas there was a reduction in accuracy across all classes, most notably in the class representing bare soil. A representation of the classified output from one area of Kasungu excluding NIR-derived variables is shown in Fig. 6, with Additional file 4 presenting the corresponding contingency table for interpolated areas only. A representation of the classified output obtained using NIR-derived variables is presented in Additional file 5. The corresponding variable importance plots are available in Additional file 6. The results are very similar for both models fitted with and without the NIR-derived variables. The variable making the greatest contribution to the classification model in both cases is the mean elevation, with mean red, blue, green and brightness also important. While the NIR-derived variables NDVI and SAVI make the greatest contribution to the classification algorithm in the second model (Additional file 6), Table 3 indicates that the inclusion of NIR-derived variables does not make any significant impact on classification accuracy, with all interpolated accuracy measures being similar when computed using a classification which includes and excludes the NIR-derived variables. For example, the overall median interpolated accuracy obtained using NIR-derived variables is marginally lower (0.907) than that obtained without using NIR-derived variables (0.912), whereas quantity disagreement increases to 22 from 21 when accounting for NIR-derived variables. There is a clear drop in all accuracy measures when considering data from the extrapolation area. Median accuracy reduces to 0.761 and 0.798 when considering overall accuracy without and with NIR-derived variables respectively whereas quantity disagreement increases to 70 and 63 respectively. This reduction in classification quality is less pronounced when considering surface water (open water or aquatic vegetation) accuracy alone compared with trying to distinguish between all 12 land cover classes.

**Table 3:** Summaries of classification accuracy

	Area	Without NIR-derived variables	With NIR-derived variables
		Median (IQR)	Median (IQR)
Overall accuracy	Interpolated	0.912 (0.878–0.916)	0.907 (0.902–0.915)
	Extrapolated	0.761 (0.754–0.770)	0.790 (0.753–0.808)
Surface water accuracy	Interpolated	0.983 (0.980–0.987)	0.983 (0.979–0.986)
	Extrapolated	0.942 (0.939–0.945)	0.935 (0.932–0.938)
Overall kappa	Interpolated	0.903 (0.894–0.908)	0.898 (0.892–0.907)
	Extrapolated	0.738 (0.731–0.748)	0.770 (0.730–0.790)
Surface water kappa	Interpolated	0.960 (0.953–0.970)	0.960 (0.951–0.967)
	Extrapolated	0.871 (0.866–0.878)	0.856 (0.848–0.863)
Overall quantity disagreement	Interpolated	21 (17–24)	22 (19–26)
	Extrapolated	70 (65–74)	63 (59–66)
Surface water quantity disagreement	Interpolated	3 (2–5)	7 (3–11)
	Extrapolated	3 (2–6)	24 (9–32)
Overall allocation disagreement	Interpolated	43 (38–46)	42 (37–48)
	Extrapolated	95 (91–101)	81 (69–107)
Surface water allocation disagreement	Interpolated	8 (6–10)	4 (2–8)
	Extrapolated	36 (34–38)	18 (12–36)
% surface water	All	18.3 (17.3–20.9)	16.6 (15.7–22.0)

Summaries are for all 12 classes (*overall)* and for *surface water* (including open water and aquatic vegetation) versus all other classes for GeoOBIA obtained with and without NIR-derived variables. Measures including *accuracy* (proportion of segments correctly classified), *Cohen’s kappa*, (0 = disagreement, 1 = complete agreement), *quantity disagreement* (different in the number of segments in each class as determined by the manual assessment and the GeoOBIA) and *allocation disagreement* (difference in the locations of segments in each class between the manual and GeoOBIA).

There is a close agreement in the percentage of the 400 m by 600 m area that is covered in surface water obtained by fitting the model with and without NIR-derived variables (without NIR: median = 18.3%; with NIR: median = 16.6%), with more variability observed in the NIR-inclusive models (Table 3). A fully manual classification of the image, independently undertaken by two researchers, resulted in a larger percentage of the area being identified as surface water (21.2 and 20.1% by researchers 1 and 2 respectively), with the union of these two outputs (‘*manual surface water classification’*) covering 22.0% of the area. This process of manual classification took approximately 2 h to complete. Using the classifications presented in Fig. 6 and Additional file 5 as an example, the intersect of the manual and GeoOBIA-derived classifications covered 67.2% (without NIR) and 61.2% (with NIR) of the union of the classifications, covering 17.1 and 15.3% of the study area respectively. Without using NIR-derived variables, 19.1% of the union was covered by the manual classification alone, with the remaining 13.7% being covered by the GeoOBIA classification along. Similarly, using NIR-derived variables, 26.8% of the union was covered by the manual classification alone, with the remaining 12.0% being covered by the GeoOBIA classification. Without extensive contemporary ground-based surveys it is not possible to determine which classification approach is more accurate, however it is clear that there are substantial differences between aerial image-based manual and automated classifications.

### Entomological surveys

During three separate field visits (June 2018, October 2019, and February 2020) a total of 326 larval sites were sampled (available through the Figshare repository [29]). During the dry periods these samples were focused along the shorelines of larger permanent water bodies (296 sites), with a mixture of 30 temporary and permanent sites surveyed during the wet season (Table 4). Both anopheline and culicine larvae were found throughout the area during each sampling period (56% of sites sampled), with the lowest proportion of positive sites found in the late dry season (76% in June 2018, 16% in October 2019, 70% in February 2020). No clear sympatry was observed between anopheline and culicine larvae in this study. For example, of the 321 sites where late stage larvae data were recorded (excluding five sites with missing data), larvae were observed in 31% (101) of samples with only 7% (24) sites containing both anophelines and culicines. In the area surrounding the Malangano site (Fig. 3) in February 2020, 177 out of 297 anopheline specimens were morphologically identified to species level, finding a predominance of *An. gambiae* sensu lato (s.l.) (87.6%, 155/177) followed by *Anopheles coustani* (8.5%, 15/177) and very few *Anopheles pretoriensis* (2.3%, 4/177) and *An. funestus* (1.7%, 3/177).

**Table 4:** Summaries of the larval sampling sites by presence/absence of mosquito larvae found

		Larvae absent N (%)	Larvae Present N (%)	Total
Sampling period				
2018 (early dry season)		31 (24)	97 (76)	128
2019 (late dry season)		141 (84)	27 (16)	168
2020 (wet season)		9 (30)	21 (70)	30
Vegetation				
Yes		148 (52)	136 (48)	284
No		33 (79)	9 (21)	42
Dominant vegetation type				
None		33 (79)	9 (21)	42
Floating		27 (61)	17 (39)	44
Submerged		30 (53)	27 (47)	57
Emerging		91 (50)	92 (50)	183
Vegetation cover				
0		33 (79)	9 (21)	42
< 1/3		56 (62)	35 (38)	91
1/3–2/3		52 (53)	46 (47)	98
> 2/3		40 (42)	55 (58)	95
Turbidity				
Turbid		74 (57)	55 (43)	129
Clear		107 (54)	90 (46)	197
Total		181 (56)	145 (44)	326

A similar table for late stage (L3–L4) larvae can be found in Additional file 7

**Fig. 7:**
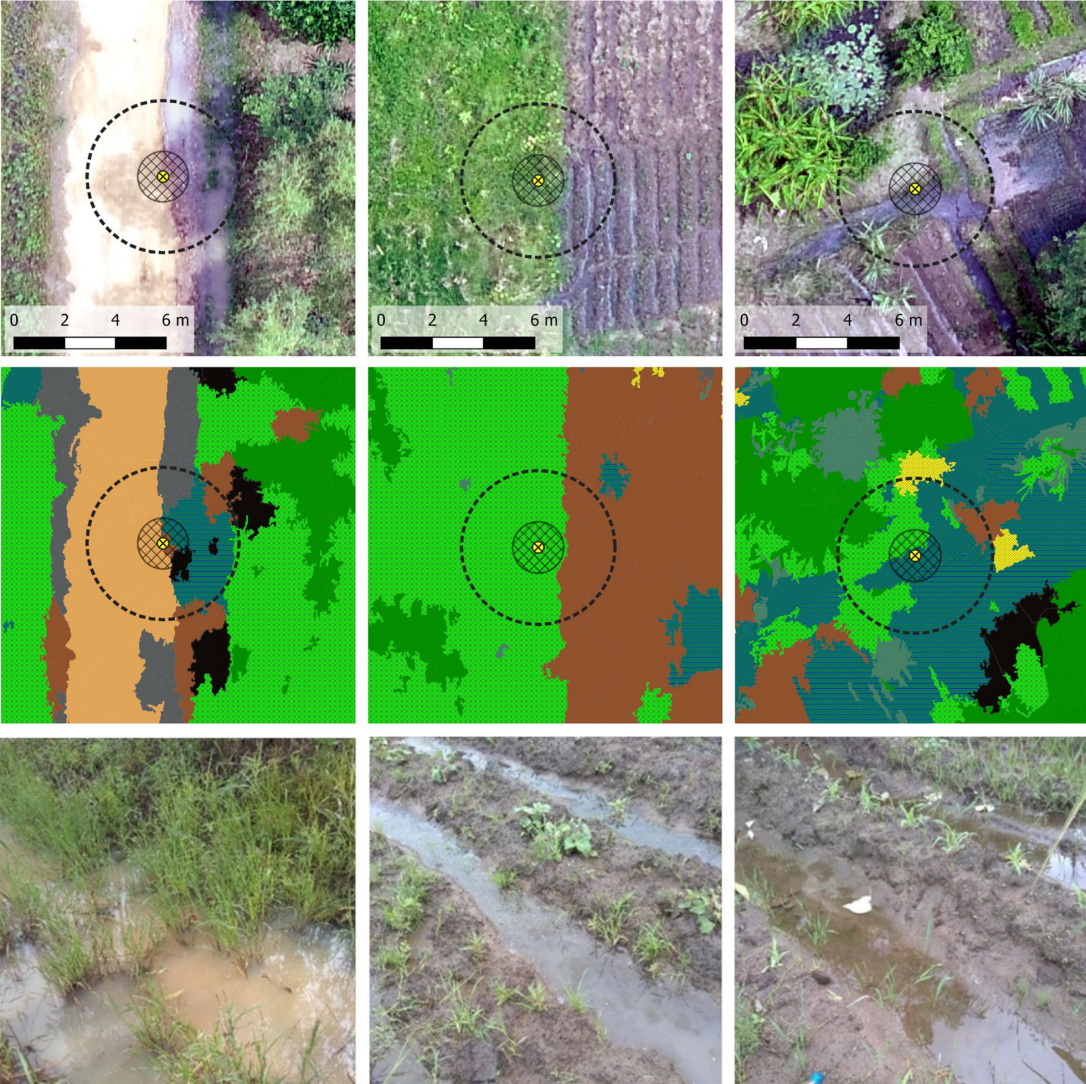
Examples of sampling sites where anopheline larvae were found. The top row indicates the precise GPS location captured using ODK (yellow circle), the expected sampling area based on these coordinates (1 m radius), and the expected accuracy of the coordinates (3 m radius), overlaid on top of the drone imagery. The middle row presents the classified imagery for these sites and the bottom row contains photographs of each site taken at the time of sampling

At each site, GPS coordinates were recorded using the ODK app, photographs were taken using a smartphone, and aerial imagery was captured (Fig. 7). Samples were taken within approximately one metre of where the researcher stood to record the coordinate, however, as GPS coordinates have an accuracy of approximately three metres it was not possible to pinpoint precisely where the samples were taken within the aerial imagery.

Using the photographs, each site was characterized according to presence/absence of larvae and sample site characteristics including dominant vegetation type (none, floating, submerged, emerging), vegetation cover (none, < 1/3, 1/3−2/3, > 2/3) and turbidity (Additional file 8). Vegetation was present in most sites sampled (284/326, 87%). Of these, 64% (183) contained emergent vegetation, 20% (57) contained submerged and 15% (44) contained floating vegetation (Table 4). Vegetation cover varied evenly across sites, with 32% having low (0–1/3) coverage, 35% having moderate (1/3−2/3 coverage) and 33% having high (> 2/3) coverage. There was an interaction between vegetation type and coverage, such that sites with floating vegetation rarely had high vegetation coverage (Additional file 9). With regards to turbidity, 40% (129) of sites were classed as turbid whereas the remaining 60% (197) were clear.

Due to the strong interaction was observed between vegetation type and coverage, when fitting the GLMMs to the presence/absence data these variables were not included in the model simultaneously, but rather explored which of the two resulted in the best fitting model with regards to Akaike Information Criterion (AIC). After counting for the effect of sampling period and site, there was a strong association between the presence of vegetation and the likelihood of any larvae (log-OR = 1.44, 95% CI [0.34, 2.67], p = 0.01), however accounting for vegetation coverage or vegetation type did not improve the model further. When considering *Anopheles* L3 and L4 larvae only (Additional file 7), the model was improved when vegetation type was considered such that larvae were more likely to be present when emerging (log-OR = 1.14, 95% CI [− 0.05, 2.51], p = 0.07) or submerged (log-OR = 1.90, 95% CI [0.51, 3.44], p = 0.01) vegetation were available, compared to sites with no vegetation. With regards to productivity, while there was variability in the abundance of larvae sampled per site (145 sites, min = 1, median = 4, max = 56), there were insufficient high productivity sites to formally explore any trends in their characteristics.

**Fig. 8:**
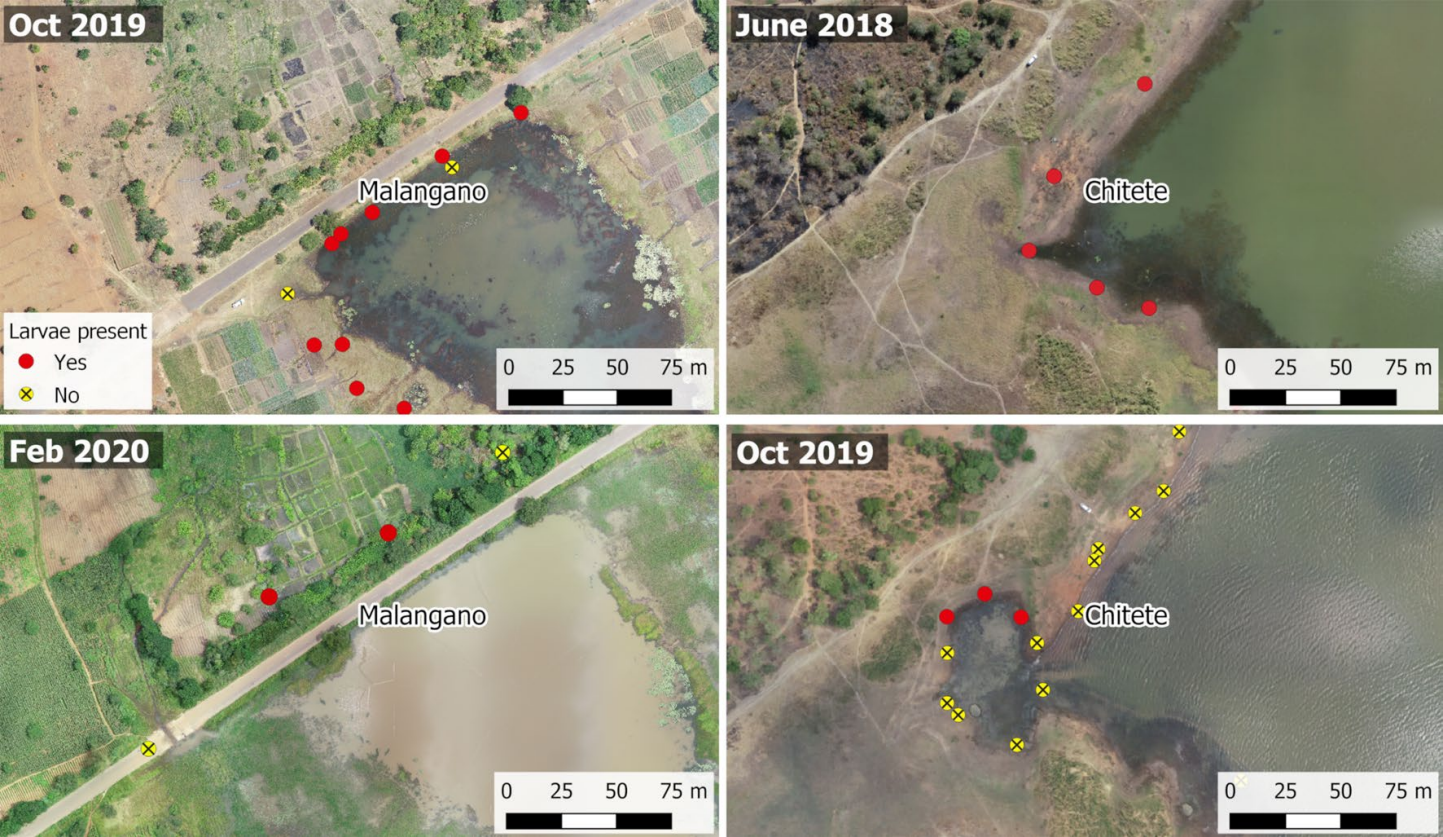
Comparisons of aerial images captured at different seasonal time points. Left images display a comparison between consecutive dry (Oct 2019) and wet (Feb 2020) seasons around the Malangano dam. Right images display comparisons between dry seasons over 2 consecutive years (June 2018, Oct 2019) around the Chitete dam

During the wet season, 10 sites were repeatedly sampled over four consecutive days, with larvae consistently observed in four sites and no larvae being found on at least one day in the remaining six sites. Due to changes in the environment it was difficult to resample the same locations across larger time scales. Temporary surface water observed in the wet season dried up even after just a few days without rain and shorelines of permanent water bodies varied substantially both between seasons and between the same season over consecutive years (Fig. 8). For example, images captured later in the dry season (October) in 2019 were wetter than those captured in the early dry season (June) in 2018 at the Chitete reservoir.

## Discussion

This study has established that established that drone-captured imagery can be used to accurately identify environmental characteristics associated with larval habitat in an area of land surrounding a manmade reservoir in Kasungu district, Malawi. Whilst this is a useful demonstration which provides valuable information on the theoretical aspects of mapping larval habitats using drone imagery, the primary objective for conducting this study was to explore the feasibility of this technology being used as part of a malaria control programmes’ toolkit. This discussion section therefore focuses on the potential benefits and practical bottlenecks of the methods applied in this paper with respect to drone image capture and drone image processing, plus the continued role of ground-based larval surveys.

### Image capturing

Image capture using drones inevitably leads to technical and skills-based challenges and a few of these have been highlighted here in the context of searching for water bodies in a rural setting. Aside from hardware and software issues, it was noted that flight experience was a key requirement to determine optimal flight times as neither the rotor or fixed wing drone could be flown in wet or windy conditions, and the study team experienced the impact of extreme weather on the hardware with multiple occasions of over-heating on warm days. This emphasized the need for extensive drone piloting training by the operator. The country’s drone regulations also need to be taken into careful consideration. In Malawi, data capture was facilitated by the relationship between UNICEF and the Department of Civil Aviation and a toolkit is currently being developed to outline the procedures that need to be followed by those wishing to fly drones for non-commercial purposes (https://www.updwg.org/wp-content/uploads/2019/12/Malawi-RPA-Toolkit-2019_Dec.-Final.pdf). While regulations vary by country, national civil aviation authorities are also requiring drone pilots to obtain accredited qualifications and seek appropriate permissions before using drones for research or humanitarian purposes. Training courses which cover both the operational and regulatory aspects of drone flying are currently quite sparse in sub-Saharan Africa and this may require future pilots to travel outside of their own country to gain the necessary experience. Should a malaria control implementer wish to use drone imagery within their programmes, they may therefore incur significant expense both in purchasing the equipment and training their staff. A solution to this would be to outsource the image capturing to qualified drone pilots operating in the area.

An additional bottleneck is the availability of hardware within the country of operation. While it may be possible to purchase off-the-shelf drones in-country, should any technical issues arise, obtaining part replacements or repairs becomes problematic and expensive. Investments are therefore being made in ‘home-grown’ drones, to support local economies, decrease the cost of equipment, and make repairs much more easily accessible. In Malawi for example, MicroMek (https://www.micromek.net/) manufacture the low-cost fixed wing drone known as EcoSoar [30], for both transporting goods and capturing imagery.

### Image processing

Processing drone imagery to create the orthomosaics is time-consuming, requires a high-spec computer and a large capacity for data storage. Therefore, to use this imagery in the field, an NMCP would require people skilled in both image capture and processing, plus access to the relevant software. These skills are not usually taught as part of standard drone pilot training, however this may change as the potential for using drone technology for humanitarian purposes is increasingly realized. For example, the African Drone and Data Academy was launched in January 2020 in Malawi to build capacity in both drone piloting and drone image processing and analysis [31].

Image classification is appealing because once the algorithm has been trained, it can simply be applied to any additional imagery captured without any or only a little additional data being required. In this analysis there was a decline in classification accuracy in areas within very close proximity to that used to train the algorithm and noted that even in this small area there were important land cover classes in the extrapolation area that did not appear in the training area e.g. red algae in the water. This challenge is likely to be exacerbated when considering areas further apart, or data collected at different time points. As more data are collected these limitations may be overcome, however in the short term, the effort required by the end-user e.g. an individual NMCP to train a classification algorithm may outweigh its benefits. In this demonstration, a geographical object-based classification approach was implemented which generated the segments and computing segment-level characteristics prior to training and applying the classification algorithm. The segment-generating process can be very time-consuming depending on the values of the segmentation parameters, the size of the area being classified and the segmentation algorithm being used [32]. While other classification techniques such as pixel-based classification may be quicker to perform, an object-based approach is the most appropriate for very high-resolution images such as that generated by drones [33]. The cost and benefits of accuracy against processing time therefore need to be considered should an NMCP wish to perform image classification in-house. The role of additional sensors in the image classification process is also unclear. In this analysis, comparisons were made between classification accuracy using imagery captured from a standard camera only (RGB) and additional imagery captured by a much more expensive NIR sensor. While the performance metrics indicated very little difference in the accuracy obtained using the two approaches, a more extensive investigation would need to be undertaken over a more environmentally diverse area before any conclusions can be drawn on the benefits to incorporating this additional technology. Further sensors such as shortwave infrared (SWIR) and thermal infrared may further improve the surface water classification accuracy [34].

A more practical solution to ‘automated’ image classification may be to persevere with the less efficient, but lower skilled task of manual classification. This task is, however, not without its drawbacks, as human error can easily miss small areas, or misclassify water containing a lot of aquatic vegetation as land and vice versa. These latter ‘*missed*’ areas are of significance, as *Anopheles* mosquitoes are generally found in water containing vegetation. The fact that there was a 10% discrepancy in the manual classification undertaken by two independent researchers, both of whom were familiar with the study area, demonstrates the fallibility in this method.

As with the drone image capture, an alternative is to outsource these activities to an organization which specializes in image processing and classification. Additionally, cloud-based computing services such as DroneDeploy’s Map Engine [35] and Google Earth Engine [36] could be used as these allow individuals/groups to harness the power of remote servers to manage and manipulate the data. This approach could facilitate the development of more automated habitat classification approaches i.e. using data from other organizations, previous field or professional expertise in remote sensing to develop classification algorithms that do not require the use of bespoke training data. The TropWet tool developed by Hardy, Oakes, and Ettritch [37] is a demonstration of this in which satellite imagery (Landsat, 30 m resolution) is automatically classified for a user-specified area and time period using a Google Earth Engine interface.

There are still practical challenges with these approaches, particularly relating to the upload of large image files to enable these processes to be undertaken remotely, however these may be preferable to the more technical challenge of managing the data in-house.

### Entomological survey

Larval surveys are an important part of the process of LSM both to confirm the species of mosquitoes found in the area, to characterize the types of surface water where larvae are likely to be found, and to monitor the progress of any subsequent intervention. Larval surveys are however a time-consuming process, particularly when undertaken during the wet season during which areas become inaccessible following heavy rains. The role of drones in LSM is not to completely remove the need for larval surveys, but to help differentiate between water bodies with respect to their potential as larval habitat and/or to differentiate sites according to their potential larval productivity.

It was noted that vegetation coverage was important when considering presence/absence of late stage *Anopheles* larvae, with coverage correlated with the type of vegetation found i.e. coverage of floating vegetation was likely to be less than that of emergent or submerged vegetation. A full understanding of the larval ecology of the local individual malaria vectors would greatly assist a targeted LSM approach aided by drone-imagery support. In south-eastern Tanzania, a basic characterization of *An. funestus* larval habitats provides support that this species occupies small spring-fed pools, permanent natural ponds and slow-moving waters each of which fall under the ‘few, fixed and findable’ paradigm [5]. In a recent study in Southern Malawi [6], *An. arabiensis* was the dominant species, with high densities being found in aquatic habitats surrounded by bare soil. A species-specific approach to identifying larval habitat using drone imagery may therefore be required, with imagery captured throughout the year to better understand the temporal dynamics of larval habitat and thereby optimize the impact of any potential intervention. These images could further be used to monitor the progress of LSM campaigns with, for example, a more accurate estimates of LSM coverage and demonstrable changes in the landscape because of habitat removal/modification. Further entomological surveillance remains pivotal to establish where and when LSM should be deployed and measure the impact of the intervention on malaria transmission potential.

## Conclusions

This study demonstrates the potential for drone imagery to be used as a tool to support the identification of mosquito larval habitat in rural areas where malaria is endemic. While this technology has the capacity to complement the more labour-intensive approach of identifying larval habitat from the ground, there are technical challenges to overcome before it can be smoothly integrated into malaria control activities. The authors believe that outsourcing the capturing and processing of drone imagery to private companies with the equipment and skills necessary to extract the required information is a more practical approach to developing equivalent skills in house. These services are becoming increasingly available in other sectors such as agriculture, forestry and environmental monitoring and there are promising developments in the African drone sector to support this local capacity. It is however important to emphasize that drone imagery should not be used to completely replace larval surveys. Instead, this technology could provide Additional information which may help to reduce the time spent finding locations to be sampled, monitor environmental changes over time and help to guide the frequency and scale of any LSM intervention, ultimately increasing its potential for success. Further consultations between experts and stakeholders in the fields of drones, image analysis and vector control are needed to develop more detailed guidance on how this technology can be most effectively exploited.

## Supplementary Information

**Additional file 1.** Locations of reservoirs around which the drone image capture and entomological surveys were focused. The area covered represents the size of the area captured in the drone imagery.

**Additional file 2.** Number of training and testing segments that were manually created for each land class.

**Additional file 3.** A description of the variables that were derived from the images captured by the drones.

**Additional file 4.** An example of a contingency table and individual class accuracy summaries obtained using a classification obtained without NIR-derived variables, applied to the interpolated area only.

**Additional file 5.** Example of a classification obtained using the random forests algorithm including NIR-derived variables

**Additional file 6.** Variable importance plot corresponding to the classification presented in Fig. 5 (without NIR) and Additional file 5: Fig. S1

**Additional file 7.** Summaries of the larval sampling sites by presence/absence of late stage mosquito larvae found.

**Additional file 8.** Examples of the vegetation types observed in the sampling sites – (from L to R) none, floating, submerged, emerging.

**Additional file 9.** Summary of larval sampling sites by presence/absence of larvae, vegetation type and vegetation cover.

## Data Availability

The entomological dataset generated and analysed during the current study is available in the Figshare repository: https://figshare.com/s/25102b084f56f41a1ca8. All drone imagery referenced in this paper is available from OpenAerialMap: (https://map.openaerialmap.org/#/33.489675521850586,-13.050633215031182,13/user/5f1fe6f357ddda00054a0647?_k=mmgy4y).
