# Supplementary material for: The application of drones for mosquito larval habitat identification in rural environments: a practical approach for malaria control?
Source: Malar J. 2021 May 31;20:244. doi: 10.1186/s12936-021-03759-2 (PMC8165685; doi:10.1186/s12936-021-03759-2)
Supplement: Supplementary file 1 — Additional file 1. Locations of reservoirs around which the drone image capture and entomological surveys were focused. The area covered represents the size of the area captured in the drone imagery. [file 12936_2021_3759_MOESM1_ESM.docx]

Table S1: Locations of reservoirs around which the drone image capture and entomological surveys were focused. The area covered represents the size of the area captured in the drone imagery.

| Name | Longitude | Latitude | Area (km^2^) |
| --- | --- | --- | --- |
| Chitete | 33.48779 | -13.05738 | 1.824 |
| Champhantha | 33.45770 | -13.05999 | 1.044 |
| Lori | 33.38845 | -13.08206 | 0.439 |
| Chimphoyo | 33.38340 | -13.11194 | 1.660 |
| Malangano | 33.43645 | -13.06844 | 1.757 |
| Farm 1 | 33.54726 | -12.99336 | 0.378 |
| Farm 2 | 33.55744 | -13.00070 | 0.439 |
| Farm 3 | 33.57166 | -12.99638 | 0.368 |
