# Supplementary material for: The application of drones for mosquito larval habitat identification in rural environments: a practical approach for malaria control?
Source: Malar J. 2021 May 31;20:244. doi: 10.1186/s12936-021-03759-2 (PMC8165685; doi:10.1186/s12936-021-03759-2)
Supplement: Supplementary file 2 — Additional file 2. Number of training and testing segments that were manually created for each land class. [file 12936_2021_3759_MOESM2_ESM.docx]

Table S2: Number of training and testing segments that were manually created for each land class.

| **Macroclass** | **Class** | **Segments** |
| --- | --- | --- |
| Water | Open water | 150 |
|  | Floating aquatic vegetation | 150 |
|  | Submerged aquatic vegetation | 150 |
|  | Emergent aquatic vegetation | 150 |
|  | TOTAL | 600 |
|  |  |  |
| Land | Trees/bushes | 250 |
|  | Grass | 250 |
|  | Bare soil | 100 |
|  | TOTAL | 600 |
|  |  |  |
| Man-made features | Iron roof (120) | 120 |
|  | Rusted iron roof (120) | 120 |
|  | Thatched roof (120) | 120 |
|  | Concrete yard/road (120) | 120 |
|  | Dirt roads/paths (120) | 120 |
|  | TOTAL | 600 |
