# Supplementary material for: The application of drones for mosquito larval habitat identification in rural environments: a practical approach for malaria control?
Source: Malar J. 2021 May 31;20:244. doi: 10.1186/s12936-021-03759-2 (PMC8165685; doi:10.1186/s12936-021-03759-2)
Supplement: Supplementary file 3 — Additional file 3. A description of the variables that were derived from the images captured by the drones. [file 12936_2021_3759_MOESM3_ESM.docx]

Table S3: A description of the variables that were derived from the images captured by the drones

| **Sensor required** | **Variable name** | **Derivation** |
| --- | --- | --- |
| **Standard RGB camera** | RGB | Values of Red, Green and Blue reflectance |
|  | Elevation | Photogrammetric methods within Agisoft Metashape |
|  | Slope | Derived from elevation |
|  | Brightness | Red+Green+Blue |
|  | Normalised difference Turbidity Index (NDTI) | (Red-Green)/(Red+Green) |
|  | Haralick texture variables | Energy, Entropy, Correlation, Inverse Difference Moment, Inertia, Cluster Shade, Cluster Prominence,  Haralick Correlation. Derived using the HaralickTextureExtraction tool within OTB |
| **NIR sensor** | NIR | Value of NIR reflectance |
| **Red and NIR** | Normalised Difference Vegetation Index (NDVI) | (NIR-Red)/(NIR+Red)* |
|  | Soil Adjusted Vegetation Index (SAVI) | 0.5(NIR-Red)/(NIR+Red+0.5) |
| **Green and NIR** | Normalised Difference Water Index (NDWI) | (Green-NIR)/(Green+NIR) |

*A modified version of this equation was used on the image captured by the Sentera sensor as per the manufacturer’s instructions
