# Supplementary material for: The application of drones for mosquito larval habitat identification in rural environments: a practical approach for malaria control?
Source: Malar J. 2021 May 31;20:244. doi: 10.1186/s12936-021-03759-2 (PMC8165685; doi:10.1186/s12936-021-03759-2)
Supplement: Supplementary file 4 — Additional file 4. An example of a contingency table and individual class accuracy summaries obtained using a classification obtained without NIR-derived variables, applied to the interpolated area only. [file 12936_2021_3759_MOESM4_ESM.docx]

|  |  |  | Reference | | | | | | | | | | | |
| --- | --- | --- | --- | --- | --- | --- | --- | --- | --- | --- | --- | --- | --- | --- |
|  |  |  | Water | Aquatic vegetation | | | Land | | | Roofs | | | Roads/paths | |
|  |  |  | Open Water | Floating | Emergent | Submerged | Trees/ bushes | Grass | Bare soil | Iron | Rusted | Thatched | Tarmacked | Untarmacked |
| Prediction | Water | Open Water | 49 | 0 | 0 | 1 | 0 | 0 | 0 | 0 | 0 | 0 | 0 | 2 |
|  | Aquatic vegetation | Floating | 1 | 54 | 0 | 1 | 0 | 0 | 0 | 0 | 0 | 0 | 0 | 0 |
|  |  | Emergent | 3 | 0 | 49 | 0 | 0 | 3 | 0 | 0 | 0 | 0 | 0 | 0 |
|  |  | Submerged | 5 | 1 | 1 | 51 | 0 | 0 | 0 | 0 | 0 | 0 | 0 | 0 |
|  | Land | Trees/bushes | 0 | 0 | 0 | 0 | 87 | 5 | 0 | 0 | 0 | 0 | 0 | 0 |
|  |  | Grass | 1 | 0 | 1 | 0 | 10 | 89 | 3 | 0 | 0 | 0 | 0 | 0 |
|  |  | Bare soil | 0 | 0 | 0 | 0 | 0 | 2 | 35 | 0 | 0 | 0 | 0 | 1 |
|  | Roofs | Iron | 0 | 0 | 0 | 0 | 0 | 0 | 0 | 40 | 0 | 0 | 0 | 0 |
|  |  | Rusted | 0 | 0 | 0 | 0 | 0 | 0 | 0 | 1 | 38 | 0 | 0 | 0 |
|  |  | Thatched | 0 | 0 | 0 | 0 | 0 | 0 | 0 | 0 | 2 | 46 | 0 | 5 |
|  | Roads/paths | Tarmacked | 0 | 0 | 0 | 0 | 0 | 0 | 1 | 0 | 0 | 0 | 38 | 0 |
|  |  | Untarmacked | 0 | 0 | 0 | 0 | 0 | 0 | 0 | 0 | 0 | 0 | 3 | 33 |
|  |  |  |  |  |  |  |  |  |  |  |  |  |  |  |
|  | User accuracy | | 0.942 | 0.964 | 0.891 | 0.879 | 0.935 | 0.856 | 0.875 | 1.000 | 0.974 | 0.868 | 0.974 | 0.917 |
|  | Producer accuracy | | 0.831 | 0.982 | 0.961 | 0.962 | 0.897 | 0.899 | 0.854 | 0.976 | 0.927 | 0.979 | 0.927 | 0.805 |
|  | Quantity disagreement | | 7 | 1 | 4 | 5 | 4 | 5 | 1 | 1 | 2 | 6 | 2 | 5 |
|  | Allocation disagreement | | 6 | 2 | 4 | 4 | 12 | 20 | 10 | 0 | 2 | 2 | 2 | 6 |

Table S4: An example of a contingency table and individual class accuracy summaries obtained using a classification obtained without NIR-derived variables, applied to the interpolated area only.
