# Supplementary material for: The application of drones for mosquito larval habitat identification in rural environments: a practical approach for malaria control?
Source: Malar J. 2021 May 31;20:244. doi: 10.1186/s12936-021-03759-2 (PMC8165685; doi:10.1186/s12936-021-03759-2)
Supplement: Supplementary file 6 — Additional file 6. Variable importance plot corresponding to the classification presented in Fig. 5 (without NIR) and Additional file 5: Fig. S1 [file 12936_2021_3759_MOESM6_ESM.docx]

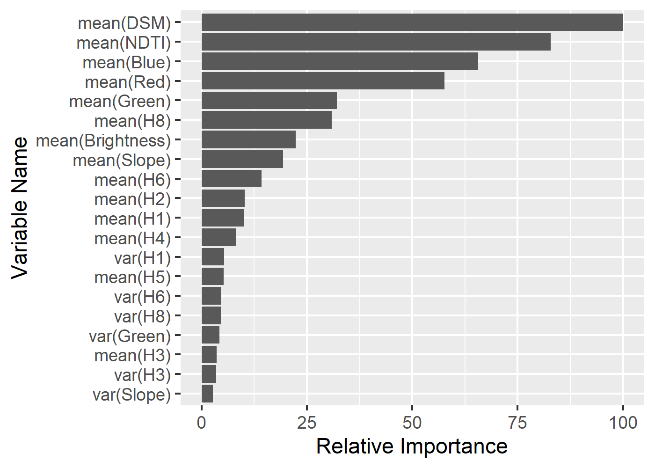

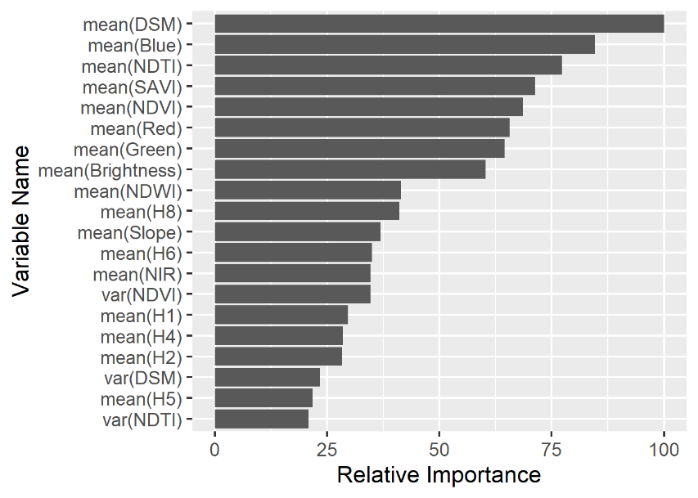


Figure S2: Variable importance plot corresponding to the classification presented in Figure 6 (without NIR) and Figure S1.
