# Supplementary material for: The application of drones for mosquito larval habitat identification in rural environments: a practical approach for malaria control?
Source: Malar J. 2021 May 31;20:244. doi: 10.1186/s12936-021-03759-2 (PMC8165685; doi:10.1186/s12936-021-03759-2)
Supplement: Supplementary file 7 — Additional file 7. Summaries of the larval sampling sites by presence/absence of late stage mosquito larvae found. [file 12936_2021_3759_MOESM7_ESM.docx]

Table S4: Summaries of the larval sampling sites by presence/absence of late stage mosquito larvae found.

|  |  | **Any L3-L4 Larvae** | | | | |
| --- | --- | --- | --- | --- | --- | --- |
|  |  | **Larvae absent** | | **Larvae present** | | **Total** |
|  |  | **N** | **(%)** | **N** | **(%)** | **N** |
| **Sampling period** | **2018 (early dry season)** | 60 | (46) | 68 | (53) | 128 |
|  | **2019 (late dry season)** | 151 | (90) | 17 | (10) | 168 |
|  | **2020 (wet season)** | 12 | (40) | 18 | (60) | 30 |
|  |  |  |  |  |  |  |
| **Vegetation** | **Yes** | 187 | (66) | 97 | (34) | 284 |
|  | **No** | 36 | (86) | 6 | (14) | 42 |
|  |  |  |  |  |  |  |
| **Dominant vegetation type** | **None** | 36 | (86) | 6 | (14) | 42 |
|  | **Floating** | 32 | (73) | 12 | (27) | 44 |
|  | **Submerged** | 36 | (63) | 21 | (37) | 57 |
|  | **Emerging** | 119 | (65) | 64 | (35) | 183 |
|  |  |  |  |  |  |  |
| **Vegetation cover** | **0** | 36 | (86) | 6 | (14) | 42 |
|  | **<**$\boldsymbol{1}/\boldsymbol{3}$ | 68 | (75) | 23 | (25) | 91 |
|  | $\boldsymbol{1}/\boldsymbol{3}$ **-** $\boldsymbol{2}/\boldsymbol{3}$ | 63 | (64) | 35 | (36) | 98 |
|  | **>**$\boldsymbol{2}/\boldsymbol{3}$ | 56 | (59) | 39 | (41) | 105 |
|  |  |  |  |  |  |  |
| **Turbidity** | **Turbid** | 136 | (69) | 61 | (31) | 197 |
|  | **Clear** | 87 | (67) | 42 | (33) | 129 |
|  |  |  |  |  |  |  |
| **Total** |  | 223 | (68) | 103 | (32) | 326 |
