# Supplementary material for: The application of drones for mosquito larval habitat identification in rural environments: a practical approach for malaria control?
Source: Malar J. 2021 May 31;20:244. doi: 10.1186/s12936-021-03759-2 (PMC8165685; doi:10.1186/s12936-021-03759-2)
Supplement: Supplementary file 8 — Additional file 8. Examples of the vegetation types observed in the sampling sites – (from L to R) none, floating, submerged, emerging. [file 12936_2021_3759_MOESM8_ESM.docx]

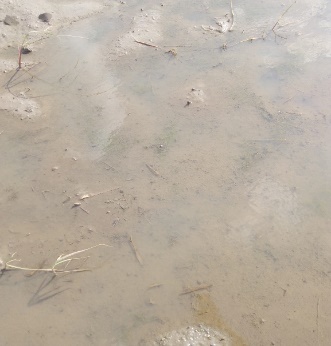

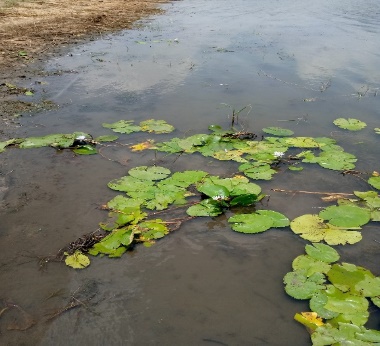

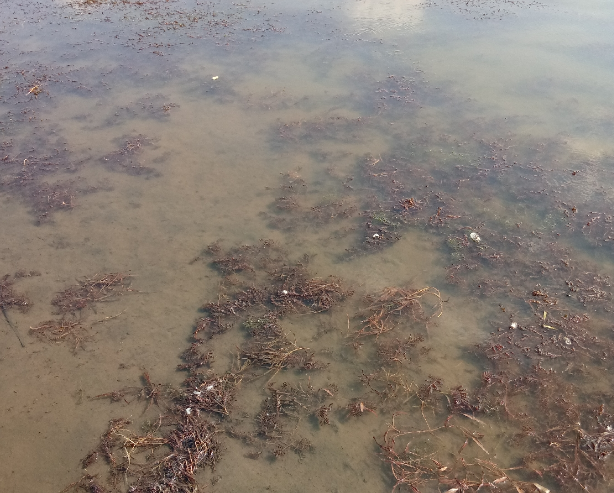

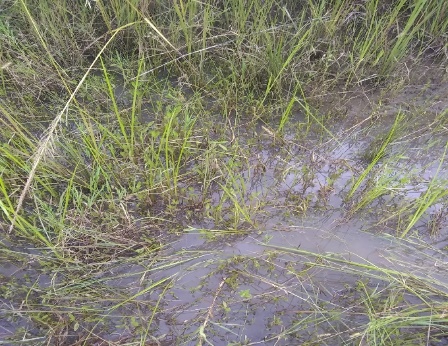


Figure S3: Examples of the vegetation types observed in the sampling sites – (from L to R) none, floating, submerged, emerging.
