# Supplementary material for: The application of drones for mosquito larval habitat identification in rural environments: a practical approach for malaria control?
Source: Malar J. 2021 May 31;20:244. doi: 10.1186/s12936-021-03759-2 (PMC8165685; doi:10.1186/s12936-021-03759-2)
Supplement: Supplementary file 9 — Additional file 9. Summary of larval sampling sites by presence/absence of larvae, vegetation type and vegetation cover. [file 12936_2021_3759_MOESM9_ESM.docx]

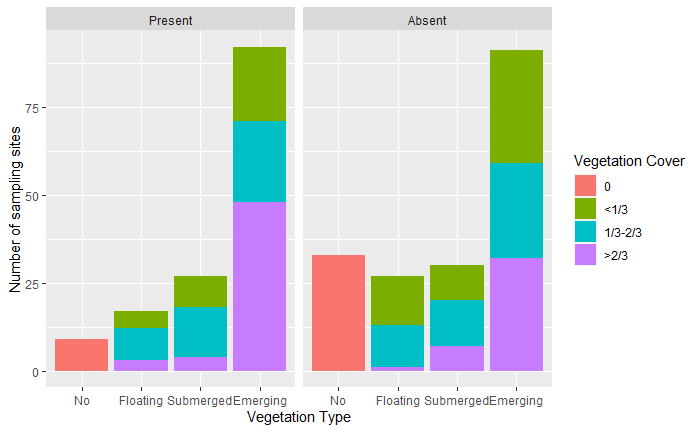
Figure S4: Summary of larval sampling sites by presence/absence of larvae, vegetation type and vegetation cover.
